# Antifungal Activity of Alpha-Mangostin against *Colletotrichum gloeosporioides* In Vitro and In Vivo

**DOI:** 10.3390/molecules25225335

**Published:** 2020-11-16

**Authors:** Huochun Ye, Qin Wang, Fadi Zhu, Gang Feng, Chao Yan, Jing Zhang

**Affiliations:** 1 Environment and Plant Protection Institute, Chinese Academy of Tropical Agricultural Science (CATAS), Haikou 571101, China; hzsyhc1312@catas.cn (H.Y.); wquiet77@163.com (Q.W.); 15901237986@163.com (F.Z.); fish0209@gmail.com (C.Y.); 2 Key Laboratory of Monitoring and Control of Tropical Agricultural and Forest Invasive Alien Pests, Ministry of Agriculture, Haikou 571101, China; 3 College of Plant Protection, Hainan University, Haikou 570228, China

**Keywords:** alpha-mangostin, antifungal activity, *Colletotrichum gloeosporioides*, spore germination, mitochondria

## Abstract

We investigated alpha-mangostin (α-mangostin, α-MG), a xanthone natural product extracted from the pericarp of mangosteen (*Garcinia mangostana*), for its antifungal activities and possible mechanism against *Colletotrichum gloeosporioides,* which causes mango anthracnose. The results demonstrated that α-MG had a relatively high in vitro inhibitory activity against *C. gloeosporioides* among 20 plant pathogenic fungi. The median effective concentration (EC_50_) values of α-MG against mycelial growth were nearly 10 times higher than those of spore germination inhibition for both strains of *C. gloeosporioides*, the carbendazim-sensitive (*CBD-s*) and carbendazim-resistant (*CBD-r*). The results suggested that α-MG exhibited a better inhibitory effect on spore germination than on the mycelial growth of *C. gloeosporioides*. Further investigation indicated that the protective effect could be superior to the therapeutic effect for mango leaves for scab development. The morphological observations of mycelium showed that α-MG caused the accumulation of dense bodies. Ultrastructural observation further revealed that α-MG caused a decrease in the quantity and shape of the swelling of mitochondria in the mycelium cells of *C. gloeosporioides*. In addition, bioassays disclosed that the inhibitory activity of α-MG on spore germination was reduced by adding exogenous adenosine triphosphate (ATP). These results suggested that the mode of action of α-MG could be involved in the destruction of mitochondrial energy metabolism. The current study supports α-MG as a natural antifungal agent in crop protection.

## 1. Introduction

*Colletotrichum gloeosporioides* is a dangerous phytopathogen threatening global plant productivity in a wide range of species, including horticultural crops (fruits, vegetables, and flowers), cereals, and grasses [1,2]. Mango anthracnose caused by *C. gloeosporioides* is one of the most dominant and destructive diseases that has emerged in the field and postharvest storage, resulting in severe rot and huge economic losses worldwide [3]. Synthetic fungicides (e.g., prochloraz, difenoconazole, carbendazim, and azoxystrobin) have been typically used as preventive and curative measures to control the infection of *C. gloeosporioides* [4]. Although chemical fungicides are effective, excessive use of these agents is now under criticism due to concerns regarding the development of fungicide resistance, food security, and environmental pollution [5,6]. In this context, the development of alternatives that are reliable, effective, and environmentally friendly is necessary. Plant metabolites is a rich source of bioactive compounds, some of which participate in plant protection against pathogen attacks [7]. During the long history of evolution, stimulated by the struggle with the pathogens, plants produce some special bioactive secondary metabolites as chemical defense barriers for combating pathogens infections [8]. These chemicals serve the plants by improving their survival fitness. These compounds have been considered as potential sources of commercial botanical fungicides and lead compounds in many studies due to their biodegradability and renewability [9,10].

**Figure 1:**
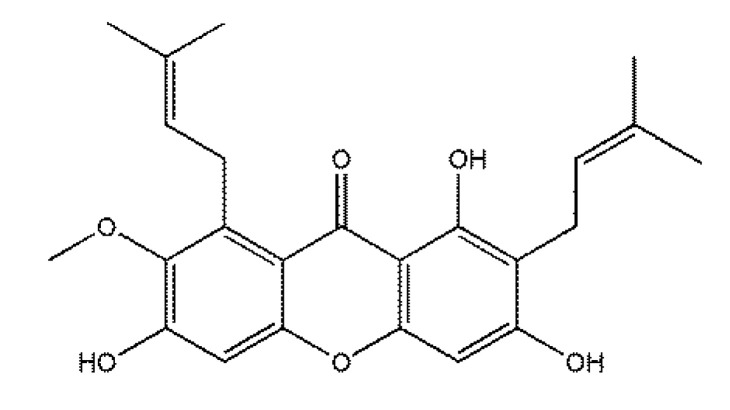
The chemical structure of alpha-mangostin (α-MG).

*Garcinia mangostana* Linn is a tropical fruit tree that is commonly cultivated in Southeast Asian regions, such as Indonesia, the Philippines, Thailand, and the Hainan province of China. Mangosteen, a fruit of *G. mangostana*, is known as “the queen of fruits” [11]. The pericarp of mangosteen has been used as an indigenous medicine for treating skin infections, wounds, and diarrhea in South East Asia [12]. Alpha-mangostin (α-MG) (Figure 1), a major xanthone compound, was first isolated from the pericarp of mangosteen [13]. Later research indicated that α-MG exists in diverse plants, including *Guttiferae, Gentianaceae, Liliaceae,* and *Leguminosae* [14]. α-MG has been demonstrated to possess multiple biological activities, including anti-inflammatory, antioxidative, antitumoral, antiparasitic, antibacterial, and insecticidal effects [15,16,17,18]. In addition, α-MG can also exert antifungal activity. α-MG and its derivatives at 1000 mg·L^−1^ demonstrated in vitro antifungal activity against three phytopathogenic fungi, including *Fusarium oxysporum vasinfectum, Alternaria tenuis*, and *Dreschlera oryzae* [19]. At a concentration of 2000 mg·L^−1^, α-MG showed a more effective fungicidal action on *Candida albicans* compared with the traditional fungicides clotrimazole and nystatin, and α-MG at 4000 mg·L^−1^ was not toxic to human gingival fibroblast for 480 min [20]. Although there are several reports on the antifungal properties of α-MG, including the antifungal spectrum using the mycelial growth method at high concentrations and structure-activity relationship [19,21], this information on antifungal mechanism is still limited.

To the best of our knowledge, there is a lack of attention towards the effects of α-MG on spore germination of phytopathogenic fungi and the related mechanisms behind the antifungal activity. In previous studies, we found that the extract from the hull of mangosteen showed a broadly antifungal activity on phytopathogenic fungi and that α-MG was a primary bioactive compound separated from the extract [22]. In this study, we investigated the antifungal activity of α-MG against 20 plant pathogenic fungi, evaluated the in vitro inhibitory activity on the mycelial growth and spore germination of *C. gloeosporioides* and the potential of α-MG to control mango leaf anthracnose, and further explored the antifungal role of α-MG against *C. gloeosporioides* using a light microscope, transmission electron microscope, and biochemical tests.

## 2. Results

### 2.1. The Antifungal Activity of α-MG against Twenty Phytopathogenic Fungi

**Figure 2:**
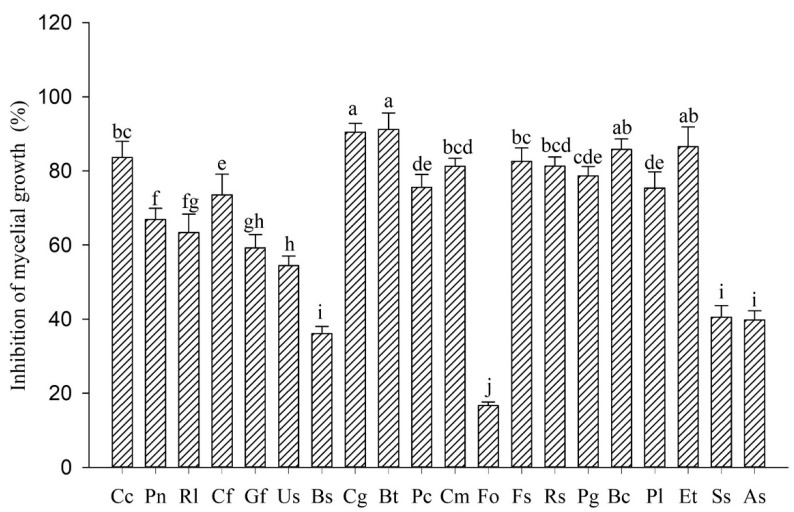
The effects of α- MG at 100 μg·mL^−1^ on the mycelial growth of 20 plant pathogenic fungi. Note: error bars represent the standard errors of the means of three replicates. Different letters within a column denote statistically significant differences (*p* < 0.05). Cc: *Corynespora cassiicola*, Pn: *Phellinus noxius*, Rl: *Rigidoprus lignosu*, Cf: *Colletotrichum falcatum*, Gf: *Gibberella fujikuroi*, Us: *Ustilago scitamine*, Bs: *Bipolaris sacchari*, Cg: *Colletotrichum gloeosporioiles*, Bt: *Botryodiplodia theobromae*, Pc: *Phytophthora capsic*, Cm: *Colletotrichum musae*, Fo: *Fusarium oxysporum*, Fs: *Fusarium semitectum*, Rs: *Rhizoctorzia solani*, Pg: *Pyricularia grisea*, Bc: *Botrytis cinereal*, Pl: *Peronophythora litchi*, Et: *Exserohilum turcicum*, Ss: *Sclerotinia sclerotiorum*, and As: *Alternaria solani*.

In total, twenty phytopathogenic fungi were employed in the antifungal bioassay using the mycelial growth rate method. The results demonstrated that α-MG (100 μg·mL^−1^) differently inhibited the mycelial growth in vitro against the tested fungi, with inhibition rates ranging from 16.67 to 91.23% (Figure 2). Among them, α-MG exhibited the highest inhibitory activity against *C. gloeosporioides* (90.38%) and *Botryodiplodia theobromae* (91.23%), and the weakest inhibitory activity against *Fusarium oxysporum* (16.67%).

### 2.2. The Effects on Mycelial Growth and Spore Germination of C. gloeosporioides

The bioassays in vitro displayed that α-MG remarkably inhibited conidia germination and mycelial growth of both carbendazim-sensitive (*CBD-s*) and -resistant (*CBD-r)* strains of *C. gloeosporioides* (Table 1). As shown in Table 1, the median effective concentration (EC_50_) values of α-MG against the mycelial growth of *CBD-s* and *CBD-r* were 73.50 and 29.09 μg·mL^−1^, respectively, while the conidia germination of the two strains were 9.06 and 2.78 μg·mL^−1^, respectively. α-MG was indicated to possess a higher toxicity to spore germination compared with mycelial growth, with EC_50_ ratios of 8.12 and 10.46 for the *CBD-s* and *CBD-r* strains, respectively.

### 2.3. Effects of α-MG on Control of C. gloeosporioides in Mango Leaves

The control efficacy of α-MG on mango anthracnose was investigated by protective and therapeutic effect tests in moisturizing plastic containers at 28 ± 1 °C and 95 ± 5% in relative humidity in the dark for 12 d. As shown in Table 2, the protective effect was significantly higher than the therapeutic effect at each concentration. At the concentration of 100 μg·mL^−1^, the protective effect of α-MG against mango anthracnose caused by *C. gloeosporioides* was 52.91%. However, the therapeutic effect only reached 43.15% at the concentration of 2000 μg·mL^−1^. In contrast, the protective effect reached 76.97% at the same concentration, and there was no significant difference with difenoconazole treated at 100 μg·mL^−1^. The results indicated that the protective effect of α-MG against anthracnose was superior to the therapeutic effect.

### 2.4. Effect of α-MG on Hyphae Morphology of C. gloeosporioides

**Figure 3:**
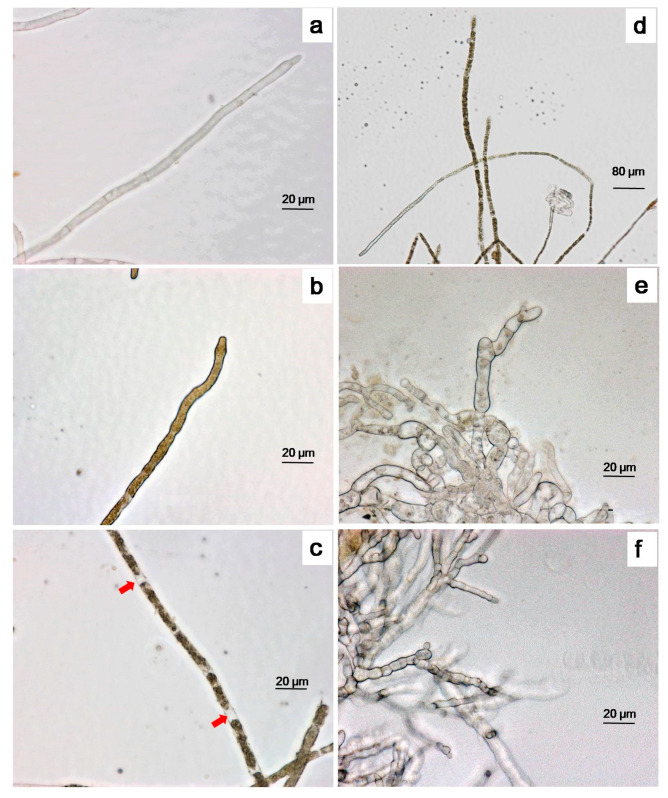
The effects of α-MG morphology of *C. gloeosporioides* in potato sucrose broth (PSB) for 24 h. Control hyphae (**a**); the hyphae treated with 500 μg·mL^−1^ α-MG (**b**–**d**); chemical control, 4.5 μg·mL^−1^ carbendazim (**e**) and 3.0 μg·mL^−1^ myclobutanil (**f**). Red arrows: intercellular cavity. Magnification: (**a**–**c**,**e**,**f**) 400×; (**d**) 200×. Bar: 20 μm.

The change of *C. gloeosporioides* hyphae morphology after treatment with α-MG was observed using a light microscope. The control mycelium grew uniformly with the edge of the hyphae being slender, linear shaped, and smooth, and the septa visible (Figure 3a). In the presence of α-MG, the dense bodies were accumulated in the cell, and the septa were not obvious or had disappeared (Figure 3b,d), while the cytoplasm in some mature hyphae was contracted to form an intercellular cavity between contiguous cells (Figure 3c). The mycelia treated with carbendazim showed distinct irregular swelling and constricting (Figure 3e). After treatment with myclobutanil, the number of hyphae branches was increased and the shape was moniliform (Figure 3f).

### 2.5. The Effects of α-MG on the Ultrastructure Transformation of C. gloeosporioides

**Figure 4:**
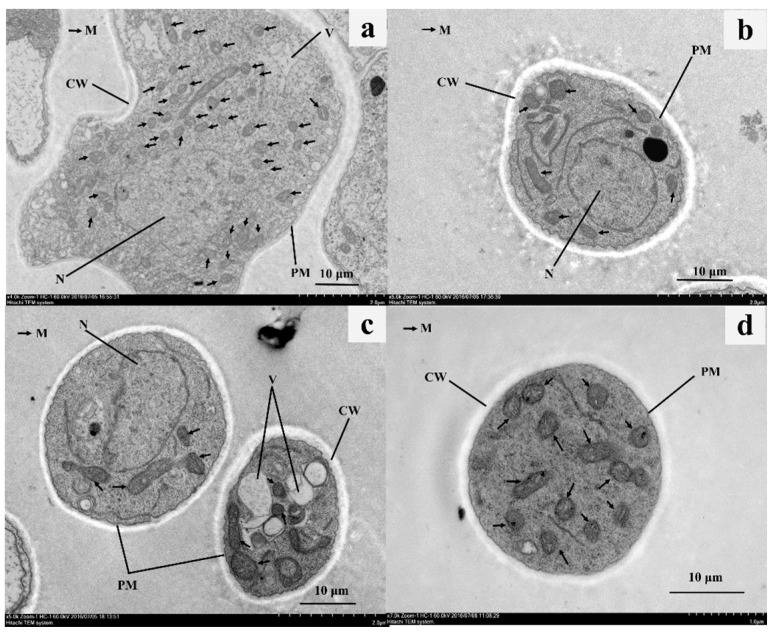
Transmission electron micrographs (TEM) of *C. gloeosporioides* treated with α-MG. The control mycelia plasma was homogenous, and the cell wall (CW), undulated plasmalemma (PM), and intracellular organelles, including the nucleus (N), mitochondria (M, black arrows), and vacuoles (V), unbroken and normal structures (**a**). Sections of *C. gloeosporioides* treated with 500 μg·mL^−1^ α-MG demonstrated tumefaction and decreasing mitochondria (**b**,**c**). *C. gloeosporioides* hypha exposed to 4.5 μg·mL^−1^ carbendazim showed disorganization of the hyphae nuclear membrane and nucleolus (**d**). Bars: 10 μm.

The cytological changes of *C. gloeosporioides* treated with α-MG were observed using transmission electron microscopy (TEM). At a concentration of 500 μg·mL^−1^, α-MG caused marked swelling in the mitochondria shape and significantly decreased the number of mitochondria, which was the most pronounced ultrastructural change observed in the hyphae. However, compared to the control (Figure 4a), α-MG treatment did not change the appearance of certain organelles, including the cell wall, cytomembrane, vacuoles, and nucleus (Figure 4b,c). In the presence of carbendazim at 4.5 μg·mL^−1^, a disorganization of the nuclear membrane and an absence of the nucleolus were observed in the hyphae of *C. gloeosporioides* (Figure 4d).

### 2.6. The Combined Effects of α-MG and Adenosine Triphosphate (ATP) on C. gloeosporioides

**Figure 5:**
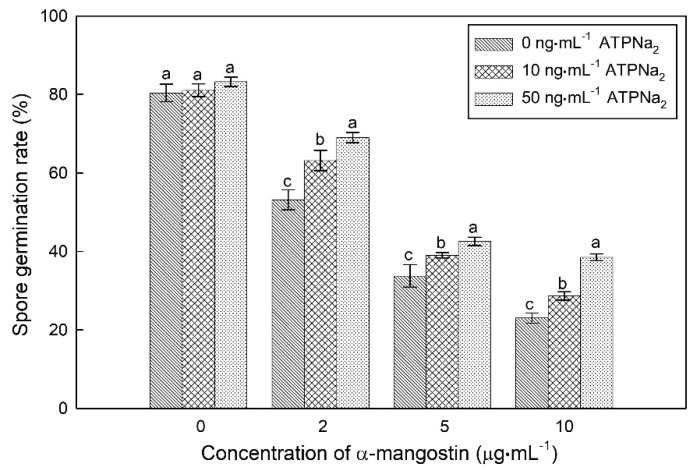
The inhibitory effect of α-mangostin in combination with ATPNa_2_ on conidia germination of *C. gloeosporioides*. Different letters on bars in the same column indicate significant differences (*p* < 0.05).

With the addition of ATP (adenosine triphosphate disodium (ATPNa_2_) as a substitute) to microplates, the spore germination rates of *C. gloeosporioides* treated with different concentrations of α-MG (2.0, 5.0, and 10.0 μg·mL^−1^) significantly increased (Figure 5). In the presence of α-MG, the germination rate of *C. gloeosporioides* supplemented with ATPNa_2_ at 50 ng·mL^−1^ was significantly higher than that at 10 ng·mL^−1^. In the presence of α-MG at 10.0 μg·mL^−1^, the germination rate was 23.01% in the absence of ATPNa_2_, whereas the rate increased to 28.65 and 38.53%, respectively, when 10 and 50 ng·mL^−1^ of ATPNa_2_ were added. These results suggested that the spore germination of *C. gloeosporioides* could be recovered to a certain extent by the addition of ATP under α-MG stress.

## 3. Discussion

α-MG has been recently explored based on its pharmacological properties (e.g., anti-inflammatory, antioxidative, and antitumoral) to human health [15]. α-MG has also been reported for use as an antibacterial, antifungal, and larvicidal agent in agricultural and food production [19]. Although the antimicrobial activity of α-MG against candida was well documented [23], there were few reports on the antifungal activity of α-MG against filamentous fungi, particularly in relation to the inhibitory activity on the spore germination of plant pathogenic fungi. In this study, *C. gloeosporioides* was found to be highly sensitive to α-MG among the tested plant pathogens. Furthermore, we found that fungal spore germination was distinctly more sensitive than the mycelial growth to α-MG at relatively low concentrations (typically 10 μg·mL^−1^), which was not regarded in previous studies. The toxicity of α-MG to spore germination was markedly higher than to mycelial growth for *C. gloeosporioides*, and the protective efficacy of α-MG was superior to its therapeutic efficacy against anthracnose from mango leaves. These findings suggest that the antifungal activity of α-MG could be predominant in inhibiting the spore germination of *C. gloeosporioides*.

The significant comparative difference of the potency of a compound against spore germination and mycelium growth can provide preliminary information on its mode of action [24]. In the fungal growth stage, mycelial growth is more sensitive than spore germination to inhibition for many fungicides, including microtubule inhibitors (e.g., carbendazim) and inhibitors of ergosterol biosynthesis (e.g., myclobutanil). However, the mitochondrial respiratory inhibitors—e.g., fluopyram (succinate dehydrogenase inhibitor (SDHI)) and kresoxim-methyl (quinone outside inhibitor (QoI))—have potent inhibitory activities on spore germination but are less active on mycelial growth [25,26,27]. Similar to mitochondrial respiratory inhibitors, α-MG exhibited inhibitory activity on spore germination, which exceeded the activity on mycelial growth.

To reveal more information regarding the antifungal properties, we observed the morphological changes of *C. gloeosporioides* after treatment with α-MG. The results showed that α-MG potentially disordered the intracellular plasma but did not affect the shape of the hyphae. Such a symptom resulting from α-MG significantly differed from the effects of carbendazim and myclobutanil, which inhibit nuclear division and ergosterol biosynthesis, respectively. This suggests that the antifungal action of α-MG could be different from the two fungicides. Further research with TEM observations showed that α-MG had profound effects on the mitochondria, including shape swelling and decreased numbers, but fewer effects on the cell wall, membrane, and nucleus. The mitochondria are the main organelles of adenosine triphosphate (ATP) synthesis, accounting for the supply of energy needed for spore germination [28]. This may explain why the inhibitory spore germination rates of *C. gloeosporioides* by α-MG could be partly relieved when exogenous ATP was supplemented.

In addition, Matsumoto et al. [29] reported that α-MG preferentially targeted the mitochondria, leading to apoptosis in eukaryotic human leukemia (HL) 60 cells. α-MG potentially interferes with the activities of energy metabolism-related enzymes, including F(H^+^)-ATPase, Nicotinamide adenine dinucleotide (NADH) oxidase, and the glycolytic enzymes aldolase and glyceraldehyde-3-phosphate dehydrogenase in *Streptococcus mutans*, a cariogenic oral bacteria (prokaryotic cells) [30]. Taken together, these findings suggest that α-MG executes an antifungal effect by targeting the fungal mitochondria and impeding the energy metabolism.

Mitochondria play a crucial role in the life cycle of eukaryotic cells, including cellar respiration; the production of ATP, NADH, and guanosine triphosphate (GTP); calcium mobilization; and the synthesis of phospholipids for membrane biogenesis [31]. ATP, which drives fundamental cell functions, is generated in the mitochondria via the tricarboxylic acid (TCA) cycle and respiratory electron transport chain pathways, which are involved in a variety of reaction enzymes and sites, including the mitochondrial membrane, cristae, five membrane protein complexes, and enzymes of oxidative phosphorylation [32]. Thus, many of them are potential targets for antifungal agents due to the disruption of the mitochondrial respiratory metabolism and shortage of energy, resulting in cell death.

Currently, eight target sites of fungicide action in mitochondria have been discovered, including succinate dehydrogenase, ubiquinol oxidase (at the quinone outside site of cytochrome bc1), and ATP synthase. Recently, a new fungicide, fenpicoxamid, which is a derivative of the natural antifungal compound UK-2A (derived from the secondary metabolites of *Streptomyces* sp. 517-02), was reported to have action at the quinone inside site of the mitochondrial respiratory cytochrome bc1 complex [33]. In our study, the inhibition of mitochondrial energy metabolism could be closely related to the mode of action of α-MG against spore germination of *C. gloeosporioides*. However, further studies are needed to identify the direct target(s) of α-MG and its role in mitochondrial functions.

In summary, α-MG exhibited strong in vitro and in vivo antifungal activity against mango anthracnose caused by *C. gloeosporioides*. Noteworthily, the inhibition of α-MG on spore germination was well above the inhibition of mycelial growth, which is similar to the efficacy of fungicides of mitochondrial respiratory inhibitors. α-MG caused the alterations of shape and quantity of mitochondria, as well as the dense body in cell of *C. gloeosporioides*. Inhibitory activity of α-MG on spore germination was reduced by adding exogenous ATP. These findings suggest that α-MG likely executes antifungal activity through the destruction of energy metabolism of the mitochondria.

The information provided here still remains insufficient, and further research should be conducted to ascertain the mode of action of α-MG to efficiently inhibit fungi. Currently, α-MG is masterly extracted from *Guttiferae* plants, with enriched xanthone compounds [34]. Recently, α-MG was successfully obtained by artificial synthesis in a laboratory [35]. Furthermore, some xanthone compounds (e.g., dimethylbellidifolin and bellidifolin-8-*O*-glucoside) have been biosynthesized in shoot cultures of *Gentianella austriaca* [36]. This provides hope for a biotechnological approach to produce α-MG in massive amounts. α-MG, as a plant-derived substance, is environmentally friendly due to its biodegradability. Based on these characteristics, α-MG could be envisaged as an antifungal agent.

## 4. Materials and Methods

### 4.1. Preparation of α-Mangostin

Fresh mature *G. mangostana* fruits were purchased from an orchard in Baoting county, Hainan province, China. The fruits were cleaned and air dried. The pericarps of mangosteen were separated from the edible pulp, dried at 40–45 °C for 60 h, and ground into a fine powder. The dried pericarp powder (1.0 kg) was soaked in 80% ethanol (4.5 L) overnight and extracted by an ultrasonic-assisted method at 50 °C for 45 min. After filtration, the liquid extraction of ethanol was evaporated at 50 °C by rotary evaporator under vacuum. The concentrated ethanol extract (77.64 g) was then suspended in water and subsequently extracted with ethyl acetate (3 × 1000 mL). The concentrated ethyl acetate extract (41.52 g) was subjected to silica gel column chromatography (45 × 600 mm), which filled with silica gel particulates (50–75 μm; Qingdao Haiyang Chemical Co., Ltd., China), eluted with gradient n-hexane/ethyl acetate (from 90:10 to 25:75, *v*/*v*). An amount of 10 μL of each eluted fraction (300 mL) was spotted manually on a thin layer chromatography (TLC) plate (silica gel GF_254_, 75–150 μm; Qingdao Haiyang Chemical Co., Ltd., Qingdao, China). The eluted fractions 76–91 were selected according to TLC profile (Rf = 0.66, eluted with hexane-ethyl acetate (70:30, *v*/*v*)). The selected fraction was freeze-dried to give a yellow compound (4.709 g) and further identified as α-mangostin (at >90% purity) by nuclear magnetic resonance spectroscopy (^1^H-NMR (400 MHz, DMSO) δ 13.73 (s, 1H), 11.02 (s, 1H), 10.83 (s, 1H), 6.80 (s, 1H), 6.35 (s, 1H), 5.18–5.17 (m, 2H), 4.02 (d, *J* = 6.4 Hz, 2H), 3.71 (s, 3H), 3.21 (d, *J* = 7.0 Hz, 2H), 1.78 (s, 3H), 1.73 (s, 3H), 1.63 (s, 6H); ^13^C-NMR (101 MHz, DMSO) δ 181.8, 162.8, 160.3, 157.4, 155.1, 154.6, 143.8, 136.8, 130.9, 130.8, 124.2, 122.9, 110.4, 110.1, 102.3, 102.3, 92.7, 60.6, 26.2, 26.0, 25.9, 21.4, 18.5, 18.2.) (Figures S1 and S2). The percent yield of α-mangostin to the dried pericarp powder was approximately 0.5% (*w*/*w*). The stock solution of α-mangostin was prepared in dimethyl sulfoxide (DMSO).

### 4.2. Fungicides and Pathogenic Fungi

Carbendazim (CBD, with a purity of 95%), myclobutanil (95% purity), and difenoconazole (95.5% purity) were obtained from Guangxi Tianyuan Biochemical Co. Ltd., Guilin, Guangxi, China. Carbendazim was dissolved in 0.1 mol·L^−1^ hydrochloric acid solution; the other agent stock solutions were prepared in DMSO.

The fungi Gibberella fujikuroi, Ustilago scitamine, Bipolaris sacchari, Phytophthora capsic, Colletotrichum musae, Fusarium oxysporum, Fusarium semitectum, Rhizoctorzia solani, Pyricularia grisea, Peronophythora litchi, Exserohilum turcicum, Sclerotinia sclerotiorum, Alternaria solani, Colletotrichum gloeosporioiles (carbendazim-sensitive or resistant), Botryodiplodia theobromae, Botrytis cinereal, Corynespora cassiicola, Phellinus noxius, Rigidoprus lignosu, and Colletotrichum falcatum were obtained from the Biorational Pesticide Research and Development Center of Northwest A&F University and Postharvest Pathology and Preservation Laboratory of the Environment and Plant Protection Institute, CATAS. Phytophthora capsica was cultured on lima bean agar medium at 28 °C. The other tested strains were maintained on potato dextrose agar (PDA) medium at 28 °C.

### 4.3. Antifungal Activity Assay

#### 4.3.1. In Vitro Effects on Mycelial Growth 

The inhibitory activity of α-mangostin against the mycelial growth of twenty plant pathogenic fungi was tested according to Zhang et al. [37]. Potato dextrose agar (PDA) was used as the basal medium for all tested fungi except for *Phytophthora capsici*, which was tested in lima bean agar medium. Aliquots of α-MG stock solutions were mixed with culture medium to a final concentration of 100 μg·mL^−1^, then poured into the petri plates (90 mm in diameter). A 4.0 mm plug of fungal mycelia taken from the periphery of a growing colony was placed into the center of the plate followed by incubation at 28 °C. Plates treated with an equal quantity of DMSO served as a normal growth control. α-MG was also tested against two strains of *C. gloeosporioides*, *CBD-s* (susceptible to CBD, with EC_50_ value of 1.86 μg·mL^−1^) and *CBD-r* (resistant to CBD, with EC_50_ value above 500 μg·mL^−1^), with the final concentrations ranging from 6.25 to 400 μg·mL^−1^. Each treatment was performed in triplicate, and the test was repeated twice.

When the mycelia growth had almost extended to the edge of petri plate, the radial growth of the fungal colonies was measured and statistically analyzed. The percentage of growth inhibition was calculated by the formula from Liu et al. [38]:

I(%)=100×[(C−T)/(C−d)]

where *I* represents the inhibition rate (%), *d* represents the diameter of fungal plugs (4 mm in diameter), and *C* and *T* indicate the average colony diameters of the fungi growth on control and treatment, respectively.

#### 4.3.2. Spore Germination Assay 

Six mycelial plugs (6 mm in diameter) cut from a colony actively growing on PSA were placed in an Erlenmeyer flask containing 100 mL of sterilized potato sucrose broth (PSB). After 5–6 days incubation at 28 °C on a whirly shaker at 180 rpm, the broths were filtered through three layers of sterile lens paper to remove mycelial debris, and centrifuged and washed with sterile distilled water three times. The concentration of conidial in suspension was adjusted to 1 × 10^6^ spores·mL^−1^ by hematocytometer determination. The conidial suspensions of *C. gloeosporioides* were mixed with equal volumes of different concentrations of α-MG (*v*/*v*, 1:1). An 80 μL aliquot of the incubation mixture from each was transferred to the wells of microtiter plates, and the plates were incubated in a moisture chamber at 28 °C in the dark for 10–12 h. The wells that were treated with an equal quantity of DMSO served as the control group.

Conidia were considered germinated when the germ tube length was equal to or greater than the conidial diameter. The germination rate was estimated by counting the number of germinated conidia for at least 300 spores. Each treatment was performed in triplicate, and the test was repeated twice with the same conditions. The inhibition rate was calculated according to the formula from Fang et al. [39]

IR(%)=100×(C−T)/C

where *IR* represents the inhibition rate, and *C* and *T* represent the average percentages of spore germination of the control and treatment, respectively. The effective concentration for 50% inhibition (EC_50_) values were estimated statistically by Probit analysis with the Probit package of IBM SPSS Statistics 22.0 software (International Business Machines Corporation, Armonk, NY, USA).

### 4.4. In Vivo Trials on Mango Leaves

Fresh and new mango (*Mangifera indica* Linn, cv. Tainong) leaves, whose color had changed from bronze to green, were collected from potted plants in greenhouses. Before the test, these leaves were rinsed with tap water twice, surface sterilized with 75% alcohol, and naturally dried. α-MG was dissolved in DMSO containing 2% Tween-80 and diluted with sterile distilled water to obtain the final concentrations of 100, 1000, and 2000 mg·L^−1^. Difenoconazole, used as the control fungicide, was dissolved in DMSO and diluted to 100 mg·L^−1^ as described above.

In the protective effect test, the front and back surfaces of the mango leaves were sprayed with α-MG and difenoconazole solutions prior to inoculation of the pathogenic fungi, respectively. Leaves treated with 1.0% DMSO solution served as a control. After the droplets dried at ambient temperature, the leaves were infected with 5 mL spore suspensions (1 × 10^6^ spores·mL^−1^) of *C. gloeosporioides* (*CBD-s* was employed in further bioassays to facilitate the comparison of antifungal agents) using the foliar spray method. Each treatment consisted of five leaves and was conducted in triplicate. Then, these leaves were enveloped with wet cotton around the petioles and placed on plastic grid plates (27 mm in height) at the bottom of each closed plastic container with water (10 mm in height) in the bottom to maintain high humidity, and finally incubated at 28 ± 1 °C in dark conditions. After 12 days of incubation, the disease index was estimated as described by Jing et al. [40]:

$$\mathrm{Disease}\;\mathrm{index}\;=\frac{\left(a\times 0\right)+\left(b\times 1\right)+\left(c\times 3\right)+\left(d\times 5\right)+\left(e\times 7\right)+\left(f\times 9\right)}{\left(a+b+c+d+e+f\right)\times 9}\times 100$$

where 0 represents no symptoms; 1, 3, 5, 7, and 9 represent the disease symptom area on the leaf <5%, 5–15%, 16–25%, 16–50%, and 51–100%, respectively; and *a*, *b*, *c*, *d*, *e*, and *f* indicate the numbers of leaves in each disease category. The controlled effect of antifungal agents against mango anthracnose was calculated by the formula:

Ce (%)=(1−T/C)×100

where *Ce* is the control effect, and *T* and *C* are the disease index of the treatment and control groups, respectively.

The therapeutic effect of α-MG on leaves was determined according to the method as described by Duan et al. [41], with minor modifications. Mycelial plugs (4 mm in diameter) from 3-day-old cultures of *C. gloeosporioides* on PSA were spotted onto the surfaces of the leaves (two plugs per leaf). These leaves were transferred to the humidifying plastic containers as described above and inoculated at 28 ± 1 °C in dark conditions. After 24 h of treatment, the leaves were sprayed with water as a control and the α-MG and difenoconazole solutions as described above. After 12 d treatment, the disease index (DI) was estimated according to Chen et al. [42]. The disease control efficacy was evaluated following the formula as described above. The experiment was performed twice with five replicates per treatment.

### 4.5. Light Microscope Observation

To evaluate the effects of α-MG on the mycelial morphology of *C. gloeosporioides*, three mycelia plugs (4 mm in diameter) from the edge of a 5-day-old colony were placed into a glass tube containing 2 mL PSB. An aliquot of α-MG stock solution was added to the test tube at a final concentration of 500 μg·mL^−1^, while carbendazim (4.5 μg·mL^−1^) and myclobutanil (3.0 μg·mL^−1^) were considered as control fungicides. The tubes without agents served as controls and were incubated at 28 °C and shaken at 120 rpm for 24 h. After incubation, the edge of the fungal colony was cut and placed on slide glass. The morphological changes of the mycelia were observed using a light microscope.

### 4.6. Transmission Electron Microscopy (TEM) Observation

Sterile and dry filter paper (0.8 cm × 5.0 cm) was placed around the edge of a 4-day-old colony in PSA plates, and we added α-MG (500 μg·mL^−1^), carbendazim (4.5 μg·mL^−1^), or 0.5% DMSO solution (used as a control). After 24 h of treatment, the mycelium pellets (1 × 1 × 3 mm) below the paper were cut for the specimens. The specimens for TEM were processed as described by Liu et al. [43], with minor modifications. The samples were pre-fixed in 3% glutaraldehyde in 0.1 mol·L^−1^ phosphate-buffered saline (PBS, pH 7.2) at 4 °C overnight. After rinsing three times with PBS for 10 min each time, the specimens were post-fixed with 1% *w/v* osmium tetraoxide solution for 2 h at room temperature and rinsed with PBS three times. The samples were dehydrated using a series of ethanol solutions in the order of concentration: 30, 50, 70, 80, 90, 95, and 100% for 15 min at each step. Afterwards, the specimens were embedded in Epon 812 and polymerize. Thin sections were cut by an EM UC6 ultramicrotome (Leica Microsystems GmbH, Wetzlar, Germany) and double-stained with uranyl acetate and lead citrate. The grids were observed using a HT7700 transmission electron microscope (Hitachi Company, Tokyo, Japan) operated at 60.0 kV.

### 4.7. The Effects of ATP on α-MG Inhibiting Spore Germination

The effects of the mixtures on *C. gloeosporioides* were evaluated according to Yan et al. [44], with minor modifications, which determined the spore germination experiments as described above. Together, α-MG with ATP (ATPNa_2_ as a substitute) were added to micro-plates containing spore suspensions of *C. gloeosporioides* to obtain the final concentrations of 10 and 50 ng·mL^−1^. The spore germination rate was measured after treatment.

### 4.8. Statistical Analyses

The data were expressed as the mean ± standard error. Duncan’s multiple range test was used to determine the significance of the differences (*p* < 0.05) between the means. The IBM SPSS 22.0 software (International Business Machines Corporation, Armonk, NY, USA) was used for the statistical analyses. The Sigma plot 12.5 program was used for graphing.

## Figures and Tables

**Table 1:** The toxicity of α-MG to two strains carbendazim-sensitive (*CBD-s*) and carbendazim-resistant (*CBD-r*) of *Colletotrichum gloeosporioides* in vitro. EC_50_: median effective concentration.CI: confidence interval.

Fungi ^a^	Inhibitory Activity	Regression Equation ^b^	*r*	EC_50_ (μg·mL^−1^) ^b^	95% CI (μg·mL^−1^) ^b^
*CBD-* *s*	Mycelia growth	y = 1.134x − 2.117	0.995	73.50	58.40–96.83
	Spore germination	y = 1.160x − 1.110	0.997	9.06	7.14–12.17
*CBD-r*	Mycelia growth	y = 1.027x − 1.503	0.992	29.09	20.75–37.86
	Spore germination	y = 0.831x − 0.369	0.919	2.78	1.37–4.20

^a^ The *CBD-s* and *CBD-r* strains of *C. gloeosporioides* were sensitive and resistant to carbendazim, respectively. ^b^ The values were statistically estimated by Probit analysis with the Probit package of SPSS 22.0 software, with a 95% confidence interval (CI) for the EC_50_.

**Table 2:** The therapeutic and protective effects of α-MG on anthracnose of mango leaves caused by *C*. *gloeosporioiles*. Different letters within a column denote statistically significant differences (*p* < 0.05).

Sample	Concentration/(μg·mL^−1^)	Protective Effect		Therapeutic Effect	
		Disease Index *	Control Efficacy	Disease Index	Control Efficacy
Control		86.67 ± 4.44	−	58.64 ± 1.07	−
α-MG	100	40.74 ± 2.57	52.91 ± 3.94 c	48.77 ± 1.07	16.80 ± 3.38 d
	1000	25.93 ± 2.57	70.14 ± 1.76 b	42.59 ± 1.85	27.39 ± 2.12 c
	2000	20.74 ± 1.28	76.97 ± 1.38 a	33.33 ± 1.85	43.15 ± 3.30 b
Difenoconazole	100	16.30 ± 1.28	79.54 ± 1.52 a	20.99 ± 2.83	64.15 ± 5.49 a

* Values are expressed as the mean of three replicates ± standard deviation.

## Supplementary Materials

The following are available online, Figures S1 and S2: ^1^H-NMR and ^13^C-NMR spectroscopy dates of α-mangostin.

## References

[1] Siddiqui Y., Ali A., Bautista-Baños S.E. (2014). Chapter 11—*Colletotrichum gloeosporioides* (anthracnose). Postharvest Decay.

[2] Piccirillo G., Carrieri R., Polizzi G., Azzaro A., Lahoz E., Fernández-Ortuño D., Vitale A. (2018). *In vitro* and *in vivo* activity of QoI fungicides against *Colletotrichum gloeosporioides* causing fruit anthracnose in *Citrus sinensis*. Sci. Hortic..

[3] Kamle M., Kumar P. (2016). Colletotrichum gloeosporioides: Pathogen of Anthracnose Disease in Mango (*Mangiferaindica* L.). Current Trends in Plant Disease Diagnostics and Management.

[4] Chiangsin R., Wanichkul K., Guest D.I., Sangchote S. (2016). Reduction of anthracnose on ripened mango fruits by chemicals, fruit bagging, and postharvest treatments. Australas. Plant. Path..

[5] Dessalegn Y., Ayalew A., Woldetsadik K. (2013). Integrating plant defense inducing chemical, inorganic salt and hot water treatments for the management of postharvest mango anthracnose. Postharvest Biol. Technol..

[6] Zhang Z.K., Yang D.P., Yang B., Gao Z.Y., Li M., Jiang Y.M., Hu M.J. (2013). β-Aminobutyric acid induces resistance of mango fruit to postharvest anthracnose caused by *Colletotrichum gloeosporioides* and enhances activity of fruit defense mechanisms. Sci. Hortic-Amst..

[7] Hüter O.F. (2011). Use of natural products in the crop protection industry. Phytochem. Rev..

[8] Yoon M.Y., Cha B., Kim J.C. (2013). Recent trends in studies on botanical fungicides in agriculture. Plant. Pathol. J..

[9] Martínez G., Regente M., Jacobi S., Del Rio M., Pinedo M., de la Canal L. (2017). Chlorogenic acid is a fungicide active against phytopathogenic fungi. Pestic. Biochem. Phys..

[10] Yoon M., Kim Y.S., Ryu S.Y., Choi G.J., Choi Y.H., Jang K.S., Cha B.J., Han S.S., Kim J.C. (2011). *In vitro* and *in vivo* antifungal activities of decursin and decursinol angelate isolated from *Angelica gigas* against *Magnaporthe oryzae*, the causal agent of rice blast. Pestic. Biochem. Phys..

[11] Wang M.H., Zhang K.J., Gu Q.L., Bi X.L., Wang J.X. (2017). Pharmacology of mangostins and their derivatives: A comprehensive review. Chin. J. Nat. Med..

[12] Jindarat S. (2014). Xanthones from mangosteen (*Garcinia mangostana*): Multi-targeting pharmacological properties. J. Med. Assoc. Thail..

[13] Schmid W. (1855). Ueber das mangostin. Eur. J. Org. Chem..

[14] Zhang X.Y., Xu Z., Lan W.J., Li H.J. (2013). Advances in studies on chemical constituents of *Garcinia mangostana* and bioactivities of xanthenones. Zhong Cao Yao.

[15] Ibrahim M.Y., Hashim N.M., Mariod A.A., Mohan S., Abdulla M.A., Abdelwahab S.I., Arbab I.A. (2016). α-mangostin from *Garcinia mangostana* Linn: An updated review of its pharmacological properties. Arab. J. Chem..

[16] Beninati S., Oliverio S., Cordella M., Rossi S., Senatore C., Liguori I., Lentini A., Piredda L., Tabolacci C. (2014). Inhibition of cell proliferation, migration and invasion of B16-F10 melanoma cells by alpha-mangostin. Biochem. Biophys. Res. Commun..

[17] Koh J.J., Qiu S.X., Zou H.X., Lakshminarayanan R., Li J.G., Zhou X.J., Tang C., Saraswathi P., Verma C., Tan D.T.H. (2013). Rapid bactericidal action of alpha-mangostin against MRSA as an outcome of membrane targeting. Biochim. Biophys. Acta.

[18] Larson R.T., Lorch J.M., Pridgeon J.W., Becnel J.J., Clark G.G., Lan Q. (2010). The biological activity of alpha-mangostin, a larvicidal botanic mosquito sterol carrier protein-2 inhibitor. J. Med. Entomol..

[19] Gopalakrishnan G., Banumathi B., Suresh G. (1997). Evaluation of the antifungal activity of natural xanthones from *Garcinia mangostana* and their synthetic derivatives. J. Nat. Prod..

[20] Kaomongkolgit R., Jamdee K., Chaisomboon N. (2009). Antifungal activity of alpha-mangostin against *Candida albicans*. J. Oral Sci..

[21] Narasimhan S., Maheshwaran S., Abu-Yousef I.A., Majdalawieh A.F., Rethavathi J., Das P.E., Poltronieri P. (2017). Anti-bacterial and anti-fungal activity of xanthones obtained via semi-synthetic modification of alpha-mangostin from *Garcinia mangostana*. Molecules.

[22] Ye H.C., Zhang J., Zhou Y., Xiao J.H., Yan C., Feng G. (2016). Pesticide Activity of the Extracts from the Pericarp of *Garcinia mangostana* Linn. Chin. J. Trop. Agric..

[23] Nguyen P.T., Falsetta M.L., Hwang G., Gonzalez-Begne M., Koo H. (2014). Alpha-mangostin disrupts the development of *Streptococcus mutans* biofilms and facilitates its mechanical removal. PLoS ONE.

[24] Slawecki R.A., Ryan E.P., Young D.H. (2002). Novel fungitoxicity assays for inhibition of germination-associated adhesion of *Botrytis cinerea* and *Puccinia recondita* spores. Appl. Environ. Microbiol..

[25] Olaya G., Zheng D.S., Köller W. (1998). Differential responses of germinating *Venturia inaequalis* conidia to kresoxim-methyl. Pest. Manag. Sci..

[26] Veloukas T., Karaoglanidis G.S. (2012). Biological activity of the succinate dehydrogenase inhibitor fluopyram against *Botrytis cinerea* and fungal baseline sensitivity. Pest. Manag. Sci..

[27] Karadimos D.A., Karaoglanidis G.S., Tzavella-Klonari K. (2005). Biological activity and physical modes of action of the Qo inhibitor fungicides trifloxystrobin and pyraclostrobin against *Cercosporabeticola*. Crop. Prot..

[28] Subikova V., Subik J. (1974). Energetic aspects of spore germination in filamentous fungi. Folia Microbiol..

[29] Matsumoto K., Akao Y., Yi H., Ohguchi K., Ito T., Tanaka T., Kobayashi E., Iinuma M., Nozawa Y. (2004). Preferential target is mitochondria in alpha-mangostin-induced apoptosis in human leukemia HL60 cells. Bioorg. Med. Chem..

[30] Nguyen P.T., Marquis R.E. (2011). Antimicrobial actions of alpha-mangostin against oral streptococci. Can. J. Microbiol..

[31] Kuhlbrandt W. (2015). Structure and function of mitochondrial membrane protein complexes. BMC Biol..

[32] Bertram R., Gram P.M., Luciani D.S., Sherman A. (2006). A simplified model for mitochondrial ATP production. J. Theor. Biol..

[33] Owen W.J., Yao C., Myung K., Kemmitt G., Leader A., Meyer K.G., Bowling A.J., Slanec T., Kramer V.J. (2017). Biological characterization of fenpicoxamid, a new fungicide with utility in cereals and other crops. Pest. Manag. Sci..

[34] Khaw K.Y., Chong C.W., Murugaiyah V. (2020). LC-QTOF-MS analysis of xanthone content in different parts of *Garcinia mangostana* and its influence on cholinesterase inhibition. J. Enzyme Inhib. Med. Chem..

[35] Iikubo K., Ishikawa Y., Ando N., Umezawa K., Nishiyama S. (2002). The first direct synthesis of α-mangostin, a potent inhibitor of the acidic sphingomyelinase. Tetrahedron Lett..

[36] Karuppusamy S. (2009). A review on trends in production of secondary metabolites from higher plants by *in vitro* tissue, organ and cell cultures. J. Med. Plants Res..

[37] Zhang J., Yan L.T., Yuan E.L., Ding H.X., Ye H.C., Zhang Z.K., Yan C., Liu Y.Q., Feng G. (2014). Antifungal activity of compounds extracted from *Cortex pseudolaricis* against *Colletotrichum gloeosporioides*. J. Agric. Food Chem..

[38] Liu F., Huang Y.J. (2011). Antifungal bioactivity of 6-bromo-4-ethoxyethylthio quinazoline. Pestic. Biochem. Phys..

[39] Fang X.L., Li Z.Z., Wang Y.H., Zhang X. (2011). *In vitro* and *in vivo* antimicrobial activity of *Xenorhabdus bovienii* YL002 against *Phytophthora capsici* and *Botrytis cinerea*. J. Appl. Microbiol..

[40] Jing C.L., Gou J.Y., Han X.B., Wu Q., Zhang C.S. (2017). *In vitro* and *in vivo* activities of eugenol against tobacco black shank caused by *Phytophthora nicotianae*. Pestic. Biochem. Phys..

[41] Duan Y.B., Ge C.Y., Liu S.M., Chen C.J., Zhou M.G. (2013). Effect of phenylpyrrole fungicide fludioxonil on morphological and physiological characteristics of *Sclerotinia sclerotiorum*. Pestic. Biochem. Phys..

[42] Chen X.L., Wang M., Yang Y., Li Y.L. (2015). Evaluation of pathogenicity of *Botryodiplodia theobromae* and resistance to stem-end rots on the main varieties of mango. J. Fruit Sci..

[43] Liu X.M., Ouyang C.B., Wang Q.X., Li Y., Yan D.D., Yang D.S., Fang W.S., Cao A.C., Guo M.X. (2017). Effects of oil extracts of *Eupatorium adenophorum* on *Phytophthora capsici* and other plant pathogenic fungi *in vitro*. Pestic. Biochem. Physiol..

[44] Yan X.J., Qin W.C., Sun L.P., Qi S.H., Yang D.B., Qin Z.H., Yuan H.Z. (2010). Study of inhibitory effects and action mechanism of the novel fungicide pyrimorph against *Phytophthora capsici*. J. Agric. Food Chem..

