# Supplementary figures and images for: Antifungal Activity of Alpha-Mangostin against Colletotrichum gloeosporioides In Vitro and In Vivo
Source: Molecules. 2020 Nov 16;25(22):5335. doi: 10.3390/molecules25225335 (PMC7696833; doi:10.3390/molecules25225335)

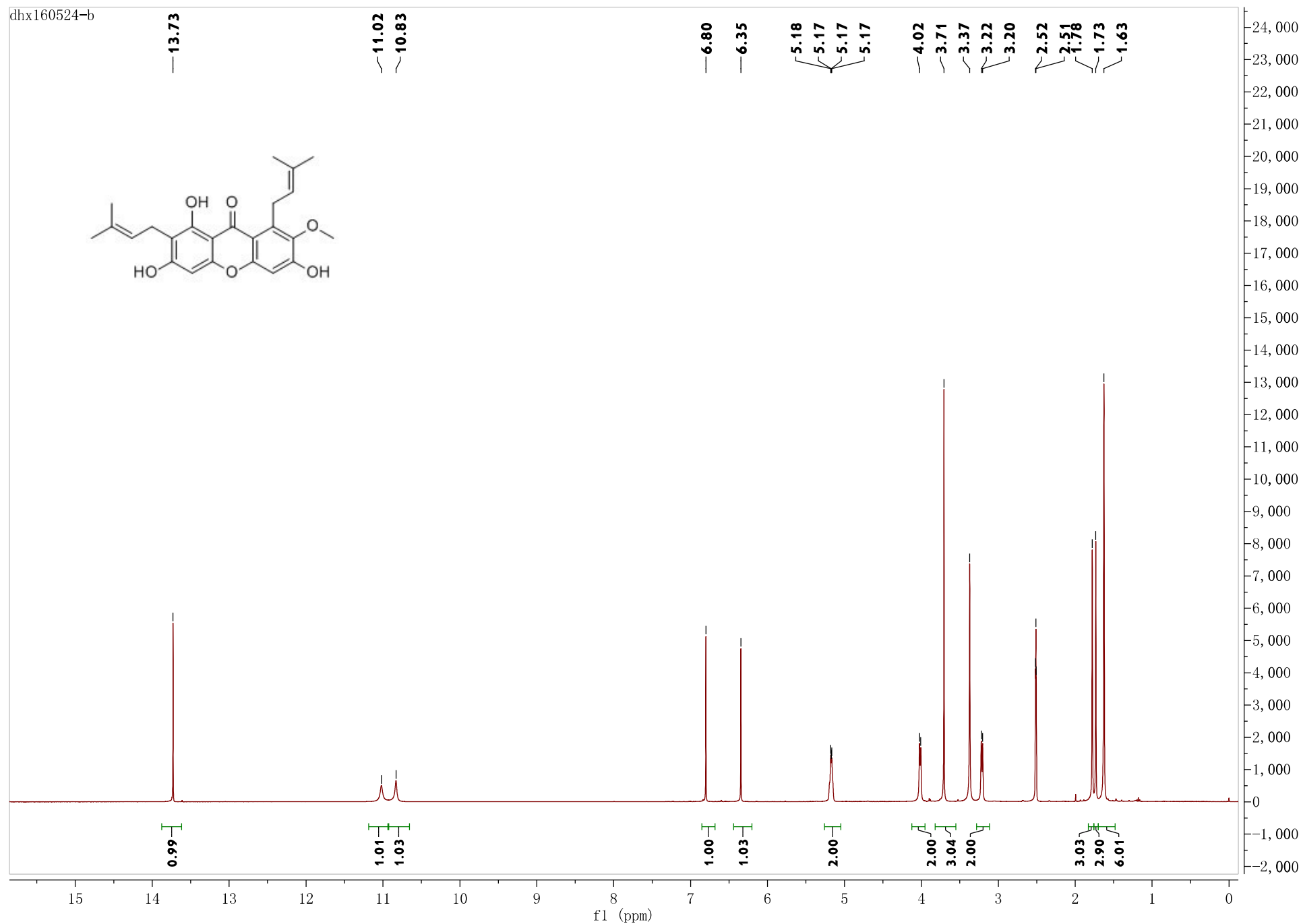

Figure S1.  $^1\text{H}$ -NMR spectrum of  $\alpha$ -mangostin.

Supplement: Supplementary file 1 [file molecules-25-05335-s001.zip › molecules-920141-supplementary/Figure S1. 1H-NMR spectra of a┴-mangostin..pdf]

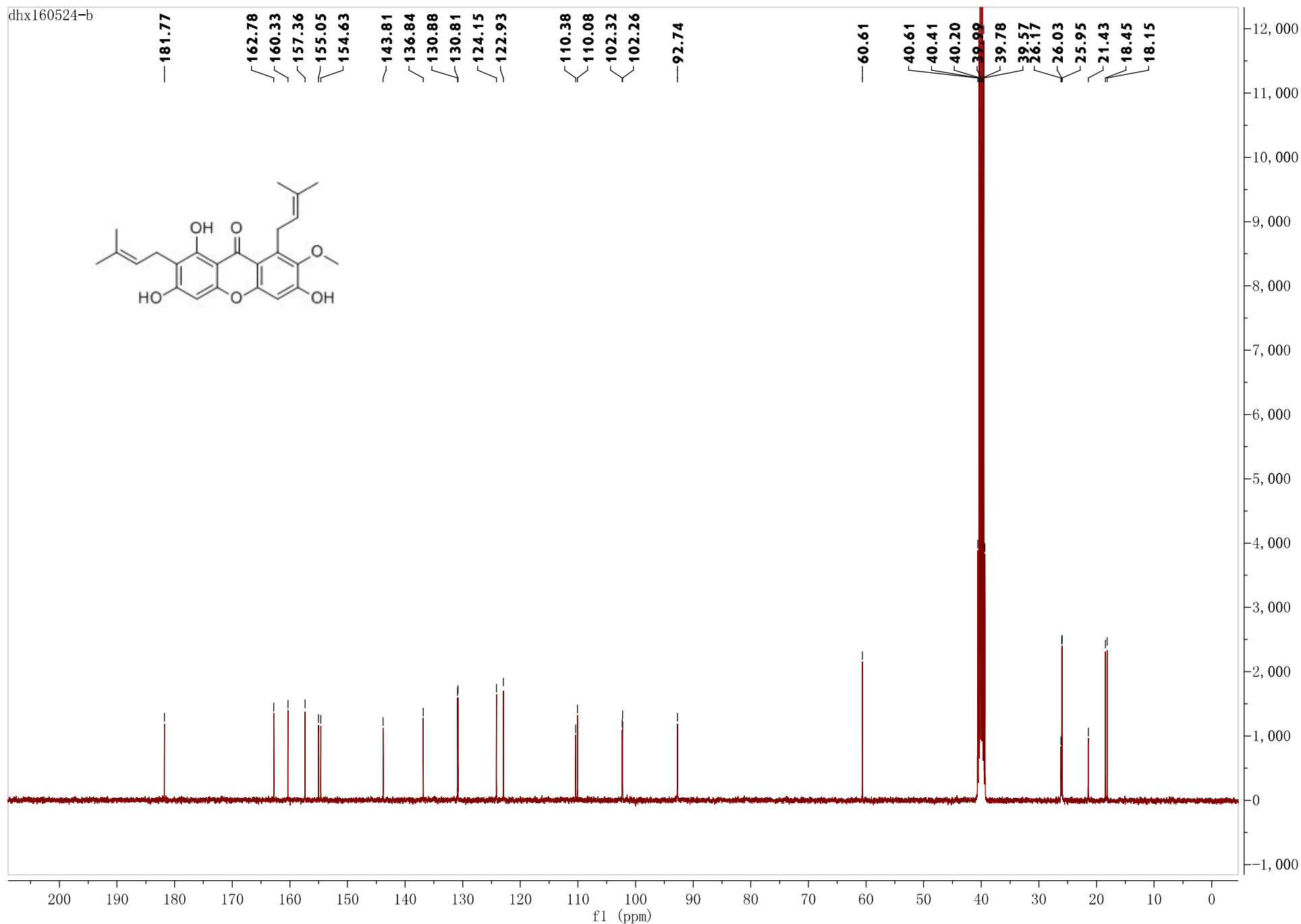

Figure S2.  $^{13}\text{C}$ -NMR spectrum of  $\alpha$ -mangostin.

Supplement: Supplementary file 1 [file molecules-25-05335-s001.zip › molecules-920141-supplementary/Figure S2. 13C-NMR spectra of a┴-mangostin..pdf]
